# Food hoarders and non-hoarders in Paridae – a cognition perspective

**DOI:** 10.1007/s10071-025-01998-3

**Published:** 2025-08-13

**Authors:** Anders Brodin

**Affiliations:** Ecology Building, Department of Biology, Lund University, Lund, S-223 62 Sweden

## Abstract

Parids are well-known birds both in Europe and North America. Despite being arboreal foragers of similar size, there is a striking dichotomy in the wintering strategies in the family. Most species are food hoarding specialists that store large amounts of winter food in autumn. A small stable group will then defend a large winter territory in which they store food. From a cognition perspective these species are spatial memory specialists with the volume of the hippocampus, a brain structure that is important for spatial memorization, correlating to the degree of specialisation for food hoarding. The wintering strategy in non-hoarding parids, the Eurasian great and blue tits, and species that are closely related to these, is very different. They are generalist foragers that have adapted especially well to anthropogenic habitats such as gardens and city parks. The great tit stands out as being especially innovative and good at observational learning, deserving its reputation as being “smartest among tits”. As the great and blue tits do not occur in North America it is possible that some chickadee populations have adapted to anthropogenic habitats as opposed to their Eurasian close relatives. The black-capped chickadee, for example, has been observed mastering foraging techniques that only the great tit does in Europe. In conclusion, there is a trade-off between two cognitive specialisations in the family with hoarding parids being spatial memory specialists and non-hoarding innovative problem solvers. The starkness of this dichotomy probably depends on that the selection for optimal foraging in winter is especially strong in small birds.

## The little bird in winter

Bird species belonging to the family Paridae, tits, titmice and chickadees, that occur in the temporal and boreal regions of North America, Europe and Asia are small passerines with a body mass between 8 and 20 g. They are either winter residents or short-distance migrants and many species spend the winter in cold regions. Despite being a rather homogenous family there are two entirely different wintering strategies with ensuing differences in cognitive abilities. In order to make a discussion of this meaningful, I will first introduce a concept known as “the little bird in winter” (Brodin 2007).

Winter is a hard time for small birds at northern latitudes. The smaller a homeothermic animal is, the larger the cooling surface will be in relation the warming body mass. This depends on the fact that surface changes on a quadratic scale whereas volume changes on a cubic one, meaning that a small animal needs to metabolize more energy per unit body mass than a large one. Hence, a 15 g parid will burn much more energy per gram body mass than a 150 g corvid during a winter night. Since parids are small birds we can assume that the selection to optimize foraging behaviour in winter has been very strong in the family.

Adding further to the problem of being small and homeothermic, winter days are short and cold at the same time as food is scarce compared to other times of the year. Food that parids prefer, such as insects and seeds may hardly be available at all. Because of this, small parids at high latitudes have to spend most of the daylight hours foraging in winter. One could then assume that they would minimize starvation risk by building up large energy reserves in their bodies.

Most birds store daily energy reserves as fat, unlike mammals which use carbohydrates for short-term storing (e.g. Lehikoinen 1987; Jenni and Jenni-Eiermann 1998). The reason is that carbohydrates bind a lot of water which makes them heavy and unsuitable for flying organisms. There are many studies, however, that show that small birds carry surprisingly small fat reserves compared to what they potentially could do (e.g. Gosler et al. 1995; Lehikoinen 1987). The reason is that there is an important second source of mortality, an ever present predation risk. The most perilous danger is an airborne predator, such as a small hawk or owl, that attacks at high speed. The counter weapon that is available to small birds is then evasive manoeuvres and speedy take-offs. The chance of escaping an attack increases the lighter the bird is, i.e. the smaller fat deposits it carries (Lima 1986; Witter and Cuthill 1993, Brodin 2007). The selective forces from the two sources of mortality are thus opposing each other. Light birds will minimize predation risk whereas fat birds will minimize starvation risk. The optimization of total mortality risk is the core of the little bird in winter problem. Small birds should hence neither minimize nor maximize fat reserves but optimize them with regards to mortality from both starvation and predation (Lima 1986; McNamara and Houston 1990, Brodin 2007).

The significance of this trade-off in parids has been demonstrated both in theoretical models and field studies (Lima 1986; McNamara and Houston 1990, Ekman and Lilliendahl 1993, Houston and McNamara 1993, Gosler et al. 1995; Brodin 2007). The human recklessness with respect to nature in the mid-1900s has provided us with strong evidence for how finely tuned this optimization is. In England, the main airborne predator of small birds is the sparrowhawk, *Accipiter nisus*. Due to pesticide poisoning sparrowhawks disappeared from much of central and eastern England in the late 1950 s (Gosler et al. 1995). Starting in 1947 thousands of great tits have been ringed and weighed in Wytham Woods outside Oxford with yearly records of body mass taken to the nearest 0.1 gram. When the sparrowhawks disappeared from Wytham great tits increased on average 0.9 gram in mass. As many birds had been captured repeatedly it was evident that this was an increase within individuals, not an effect of natural selection over years (Gosler et al. 1995).

This is a considerable increase for a bird weighing 17–20 g, around 5% of its lean body mass. With no airborne predators around, the great tits could afford to carry more fat thereby decreasing the risk of starvation. In the 1970 s, when the sparrowhawk population recovered the great tits went back to their previous match weight by decreasing on average 0.9 gram. Even more convincing was that the authors included ringing data from all over the UK from BTO, the British Trust for Ornithology. They showed that this pattern occurred in all areas where the sparrowhawks had disappeared but that there was no such effect in regions where they remained in healthy populations (Gosler et al. 1995).

The fact that parids birds carry much less fat deposits than they are able to is another line of evidence (Lima 1986; Lehikoinen 1987) and this has been demonstrated also in hoarding species. In winter, willow tits *Poecile montanus* form small flocks, usually of four individuals, that defend a large territory against other conspecifics. The flocks are dominance-structured with a strong hierarchy. By mounting a perch on top of an electronic balance near bird feeders Ekman and Lilliendahl (1993) could weigh the same individual birds repeatedly. Even though dominants have prior access to food, these carried around 0.5 g less fat than subordinate individuals. They could afford to do this since the priority gives them a more predictable access to food. This change is of the same relative magnitude as the one in the great tits, since willow tits weigh 10–12 g. When the authors removed the dominants the subordinate birds decreased their fat reserves to the same levels as the previous dominants had carried (Ekman and Lilliendahl 1993).

This shows that small birds at northern latitudes maintain a finely tuned level of fat reserves in winter and that winter foraging strategies are under strong selection for optimality.

As nervous tissue is very energetically expensive it will be economical to maintain a brain volume that is as small as possible. If one part of the brain is enlarged, for example the hippocampus in food hoarding species, it is possible that this will have a negative effect on other parts of the brain. For small energetically stressed birds such as parids, this may be a reason for a trade-off in cognitive abilities. Some species may then develop better spatial memory abilities whereas others instead may become more innovative and better observational learners.

## The Paridae

I will henceforward restrict myself to parid species in North America and northern Eurasia. These are arboreal forest dwellers although some species have adapted well to anthropogenic habitats. They are very active foragers, usually on the move while searching for food. With their strong legs it is not unusual that they will be hanging upside down when foraging requires this.

Most species are known for their food hoarding behaviour. The most industrious hoarders, such as the willow tit and the Siberian tit, in North America known as the gray-headed chickadee, *Poecile cinctus* may store 50 000 food items or even more, per individual and year (Haftorn 1956; Pravosudov 1985; Brodin 1994). Other species in which natural hoarding has been studied systematically in the field include the coal tit *Periparus ater* (Haftorn 1956), the crested tit *Lophophanes cristatus* (Haftorn 1956; Brodin et al. 1994), the marsh tit (Haftorn 1959), the varied tit *Poecile varius* (Higuchi 1977) and the black-capped chickadee *Poecile atricapillus* (Brodin 2005).

Winter residency is a necessity for long-term food hoarding to make sense and most hoarding parids maintain a large territory in which they remain the whole winter. Stable and tightly knit flocks consisting of 2–8 individuals will defend the territory against conspecific intruders (Ekman 1989). There is a strict dominance hierarchy in these resident flocks in that males dominate females and older birds younger (Ekman 1979; Ekman and Askenmo 1984; Hogstad 1988, Ekman 1989; Brodin 1994c).

Large-scale food hoarding may seem a superior strategy when one considers the energy requirements for a small bird in a cold winter climate. The two most well-known European parids, the great tit and the blue tit *Cyanistes caeruleus*, however, exhibit a very different wintering strategy. Thery do not store food at all and move around a home range that is much larger than the winter territories of the hoarding species. Flocks are large and loose in the sense that individual birds can leave a flock and join another. There is a dominance hierarchy also in these flocks but in general, conspecifics are tolerated (Ekman 1989). The hoarding species will usually concentrate on a few species of seeds and insects (Haftorn 1956; Pravosudov 1985; Brodin 1994a) while the blue and great tits are generalists that will eat almost anything edible they can find (Gosler and Clement 2007).

## Food hoarders

My own interest in food hoarding parids started when I was an undergraduate student in biology at Stockholm University. During a course in ethology at a field station a recent issue of Scientific American was laying on a coffee table. On the front cover there was a picture of a marsh tit with a plastic cup covering one of its eyes. I started reading the featured article, *Memory in food hoarding* birds by Sara Shettleworth (1983), and was captivated. And I have been ever since. Some years later I started a PhD at this university working on food hoarding behaviour in willow tits in a nearby national park. For North American readers *willow tit* may sound as some exotic foreign species, but it is so similar to the most well-known North American parid, the black-capped chickadee, so that they were considered as the same species until the mid-1950-s (e.g. Haftorn 1956).

Most work on cognition in hoarding parids has been performed on various aspects of their spatial memory, frequently with correlations to the hippocampus, a brain structure that is involved in the formation of spatial memories. The underlying assumption is that food hoarders use memory to retrieve caches, an assumption that is well-substantiated (Sherry et al. 1981; Sherry 1984; Hitchcock and Sherry 1990; Brodin and Kunz 1997). Food storing parids are scatter-hoarders that store each food item, for example a small seed, in a separate position. The caches are dispersed over a relatively large year-round territory and protected against theft by concealment or crypsis rather physical defence. An ability to remember the exact positions caches will make retrieval effective compared to if they are stored without memorization of positions.

It has been demonstrated that food hoarding bird species have relatively larger hippocampi than non-hoarding relatives (Krebs et al. 1989; Sherry et al. 1989). More specifically in parids, it has been shown that highly specialized hoarders have larger hippocampi than hoarders of intermediate specialization, and that these in turn, possess larger ones than non-hoarders (Hampton et al. 1995; Healy and Krebs 1996; Lucas et al. 2004). As larger birds have larger hippocampi simply because they are large, it is the relative hippocampal volume that is of interest here.

To calculate relative hippocampal volume correlations have been made both to body mass and telencephalon volume (Hampton et al. 1995; Healy and Krebs 1996; Brodin and Lundborg 2003; Lucas et al. 2004) and I have previously advocated the latter alternative. The reason is that many factors that are not related to the brain such as body fat deposits can cause variation in body mass, even in one day in the same individual. In winter a small parid can gain almost 10% of its lean body mass in fat in one day and the level may vary almost this much also with changes in dominance rank (Ekman and Lilliendahl 1993). Furthermore changes between seasons, like winter fattening, will also be of this magnitude (Lehikoinen 1987). Since the hippocampus is an integral part of the brain confounding external factor should affect these structures in a similar way. This suggests that hippocampal volume relative to telencephalon should be the preferrable measure of relative hippocampal volume.

**Fig. 1:**
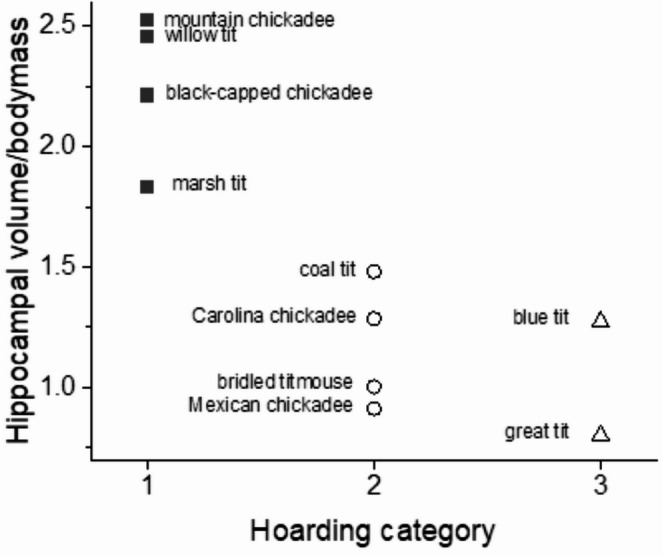
Hippocampal volume relative to body mass in parids. Hoarding categories are (1) Highly specialized hoarders (black squares), (2) Hoarders of intermediate specialization (open circles) and (3) Non-hoarders (open triangles). For definitions of these categories and references to the original publications of data prior to 2004 see Brodin and Lundborg (2023). Data on American poecilids are by courtesy of Vladimir Pravosudov (black-capped and mountain chickadees) and Jeffery Lucas (Carolina chickadees) (Roth and Pravosudov 2009; Freas et al. 2012)

LaDage et al. (2009), however, pointed out that telencephalon volumes have been measured with two methods that will give very different results, frequently without specification of which method that has been used. Hence, volume relative to body mass should still be the more usable measure. Since the studies cited in the paragraphs above were published additional data has been collected in several species. Thus I think that Fig. 1 is the most reliable depiction so far of relative hippocampal volumes in food hoarding parids.

The volume measured as mm^3^ hippocampus tissue * g^−1^ body mass was 1.04 ± SD 0.33 in non-hoarders, 1.17 ± SD 0.26 in hoarders of intermediate specialization and 2.25 ± SD 0.31 in highly specialized hoarders (Fig. 1). An ANOVA shows that the difference is clearly significant (*n* = 10, df_b_ = 2, *F* = 17.6, *p* = 0.0015). This is strong support for the assumption that food-hoarding parids are spatial memory specialists with ensuing morphological adaptations in the brain.

Most studies on cognitive abilities in hoarding parids have concerned various aspects of their spatial memory abilities or cache protection strategies. In a classic experiment Sherry et al. (1981) provided conclusive evidence that marsh tits use visual spatial memory when they retrieve caches. The authors allowed the birds to store sunflower seeds in an indoor arena with one of the eyes covered by an opaque plastic cup. When the birds later were allowed to search for the cached and invisible items, they did this either with the cup remaining on the same eye, or with the cup transferred to the opposite eye. With the cup remaining on the same eye they retrieved with normal success, but when it had been transferred to the other eye, retrieval success went down to random search level. The experimenters took advantage of the fact that small passerines have a complete crossing of the right and left optic nerves in the optic chiasma meaning that the visual impressions from one eye go to the opposite brain hemisphere. This inspection seems especially important since parids will typically turn their head 90° and make a close inspection of the cache with one eye only directly after making a cache, apparently a memorisation process (Gibb 1954; Brodin and Urhan 2014a, b).

It has been demonstrated that black-capped chickadees do not return to caches they previously have emptied or previously have found emptied and that they remember which type of food that they have stored in a cache (Sherry 1984). In order to protect their stores mountain chickadees *Poecile gambeli* preferred to cache out of view of red-breasted nuthatches *Sitta canadensis*, a potential cache thief, but appeared not to mind being observed by dark-eyed juncos *Junco hyemalis*, which do not pose such a threat (Pravosudov 2008).

## Non-hoarders

In contrast to the North American parids the two most well-known Eurasian parids, the great and blue tits, are non-hoarders (Ekman 1989). Both these are well-studied though, with the great tit being the world’s most studied wild bird species with over 70 000 published papers mentioning it according to Google scholar. Both species are successful colonizers of anthropogenic habitats such as suburban gardens, city parks and university campuses.

Especially the great tit, but to some degree also the blue tit perform better than the hoarding species in tasks are useful when it comes to adaptation to anthropogenic habitats, for example problem solving, impulse control and observational learning (Sasvári 1979; Isaksson et al. 2018; Urhan et al. 2023). If these two species are compared it seems like the great tit is more innovative than the blue tit, an assumption that is supported by the fact that they perform better when both species are tested in the same cognition experiments (Sasvári 1979; Isaksson et al. 2018; Urhan et al. 2023).

The most well-known anthropogenic behaviour in parids may be the opening of milk bottle caps that started in southern England in 1921. In these days milk bottles were covered by waxed carboard caps and left outside the front door when they were delivered to people’s houses in the morning. Milk was not as effectively homogenised as nowadays, meaning that there was a layer of fat cream in the top of the bottle, very desirable food for parids. At the end of the 1940-s this behaviour was common in great and blue tits over most of the UK (Fisher and Hinde 1949). By comparing dispersal distances of young great and blue tits with the speed of spread of the behaviour across the UK the authors concluded that it had spread faster than expected if observational learning had been the only mechanism behind this. To explain this they suggested that cap-opening had spread by a combination of observational learning and independent innovations in new locations. They suggested that the behaviour had been invented de novo in at least 89 different places in the UK. From each point of origin, it had then spread in different directions by observational learning in the local tit populations. Analysing the data of Fisher and Hinde, Lefevbre (1995) supported the hypothesis of several points or origin. He also concluded that speed of spread accelerated over time.

Such spread of behavioural traditions among animals has frequently been called cultural transmission by learning (Sherry and Galef 1984; Lefevbre 1995, Lefebvre and Boogert 2010). Sherry and Galef (1984) hypothesized that the local spread of the behaviour could be caused by great and blue tits encountering bottles that already had been pierced open by other birds rather than by observational learning. In an experiment on black-capped chickadees they showed that this mechanism also could work but could not conclude if it was important or not. I think that their proposed scenario could occur, but the evidence for the significance of cultural transmission by learning in parids is by now overwhelming (Sasvári 1979; Aplin 2019; Aplin et al. 2013, 2015). Furthermore, birds are lactose-intolerant and do not drink milk. This means that opened bottles where the cream already is taken, is not attractive to them.

Even if there were more observations of blue tits than great tits opening bottles I believe that the original inventors in most cases were great tits. There are several reasons for this. First, this type of milk delivery has also occurred in Denmark, the Netherlands and Sweden. In these countries home deliveries of milk started much later and the behaviour had thus not had a long time to spread when Hinde and Fisher (1951) summarized reports from outside the UK. In total, there was one single report, from Sweden, of a blue tit opening a bottle cap but not a single one from the other two countries. There were many reports, however, from all three countries of great tits doing it. In Copenhagen, great tits were even considered to be “milk-bottle pests”.

I have collected observations of interesting foraging behaviours by the public in calls in Swedish media since 2014. Most are about innovative foraging behaviour in great tits, but I have also received some reports on this in blue tits. Some of these reports are very brief or of low quality while others seem detailed and accurate. Here I will consider only the 90 reports that seem most reliable. I will only discuss unequivocal behaviours such as drumming on beehives or tapping at windows, whereas I do not consider behaviour that rely on more subjective considerations like the gaze direction or believed intentions of the birds.

The most frequently reported behaviour is about pecking or drumming on windows (*n* = 20). All over Sweden great tits have learnt to peck on windows in order to get a refill when bird feeders are empty. A couple of other behaviours can be merged under the label *elaborate methods to capture large insects* (*n* = 6). In some places great tits have become skilled in catching bees in winter. When it is below freezing, bees don’t fly. Great tits may then come in flocks to bee yards and stir up the colonies, sometimes almost to a frenzy. By drumming on the front of the hives they will lure the bees to come walking out, being easy prey for hungry birds (Natur & Trädgård 2017; Johnsson and Brodin 2019). In other places great tits may hide in early blooming fruit trees waiting for incoming bumblebees (Goulson 2014; Johnsson and Brodin 2019). At this time of the year, it is the queens that are foraging before they start new colony. With a quick peck great tits will make a hole at the posterior end of the abdomen, at the position where the sting usually is, and pull out the interior of the bee. The bee exoskeleton may look intact even though it almost is an empty shell. In some places there will be small piles of bumblebee shells under richly blooming trees. In other places great tits will attacking large butterflies when trees or shrubs that these prefer are flowering. In this case, there will be butterfly wings, for example of peacock butterflies *Aglais io*, piling up under positions where the birds perch while eating (Andersson and Brodin In press).

I have received three reports of great tits (and one of a blue tit) giving false alarm calls at feeders in order to scare other birds off and monopolize the food. Above I discussed the imminent danger to small birds from an incoming airborne predator that attacks in speed. On such an occasion all small birds talk the same language. A high-pitched *seee* will make other birds at the feeder take off in panic. If other birds learn that a bird is giving fake alarm calls this should not work any longer. There in a YouTube clip from England showing a great tit doing precisely this (see Youtube^1^). Still, this behaviour is rare and that, in combination with the danger of ignoring a true such call is probably the reason for why it can remain and work despite such cheating (Brodin 2024).

Impressive foraging behaviours are not the only evidence of the great tit’s ability to learn and adapt. Together with the blue tit it has become one of the commonest birds in anthropogenic habitats such as city parks and villa gardens. Great tit populations are denser in such habitats than in their original forest one, the deciduous temperate forest. One could believe that they would be stressed or affected by pollution in anthropogenic habitats. Even though populations are denser in urban habitats, however, the level of corticosterone is lower in urban than in forest populations (Brodin and Watson 2023). Furthermore, the great tit has colonized the northern taiga forest in Fennoscandia during the last century, a habitat where they previously did not exist. This also has an anthropogenic twist. In winter when it becomes really cold, they will leave the forest and go to the nearest human houses with bird feeders.

## Comparisons of cognition in hoarders and non-hoarders

In a classic study Sasvári (1979) trained captive great tits, blue tits and marsh tits to collect mealworms from a hole in a wooden box in an aviary. The hole was covered with a linen cloth that acted as a curtain covering the hole. In order to get to the mealworms a bird had to fly to the box, lift the curtain and stick its head into the hole. First, he trained individuals of all three species to act as demonstrators. He started with the hole uncovered and then gradually covered it until the mealworms were no longer directly visible and the birds had learnt the procedure. He then allowed naïve individuals to sit in a cage and observe a trained bird taking mealworms out of the hole. Regardless of the species identity of the demonstrating individual great tits learnt the task much quicker than blue and marsh tits and the differences were highly significant. This means that great tits were better at understanding what individuals the two other species were doing than their own conspecifics. Blue tits performed slightly better than marsh tits, but the difference was not significant (Sasvári 1979).

Suggesting that self-control is a good measure for general cognitive ability, MacLean et al. (2014) tested 36 species of birds and mammals in the so called transparent cylinder test. In this test, an animal is allowed to approach a transparent tube or cylinder with its openings positioned perpendicularly to the animals direction of approach. Inside the tube a desirable piece of food is visible to the animal. If it tries to take the food through the wall this counts as a failed attempt. If it instead moves to the opening of the cylinder and takes the food this way it counts as a successful one. Kabadayi et al. (2017) tested three species of corvids, adding to the data set of MacLean et al. (2014).

The success in these studies was measured as number of correct choices out of the ten first attempts. Not surprisingly, apes and large corvids in the *Corvus* genus performed best, between 90 and 100%. In my own lab we have tested two species of parids, the great tit and the blue tit. The great tits succeeded in 0.68 ± SD 0.17 and the blue tit in 52.0 ± SD 0.17, a significant difference (Urhan et al. 2023). From Can Kabadayi (Kabadayi et al. 2017) however, we learnt that the ravens, that performed at 100%, were tame birds that had had access to cut-open transparent plastic bottles as toys in their aviaries before the experiment. Our great and blue tits were wild birds that only had been given a couple of days to get used to being captivity. Most likely, our birds had little or no experience of transparent cylinders.

**Fig. 2:**
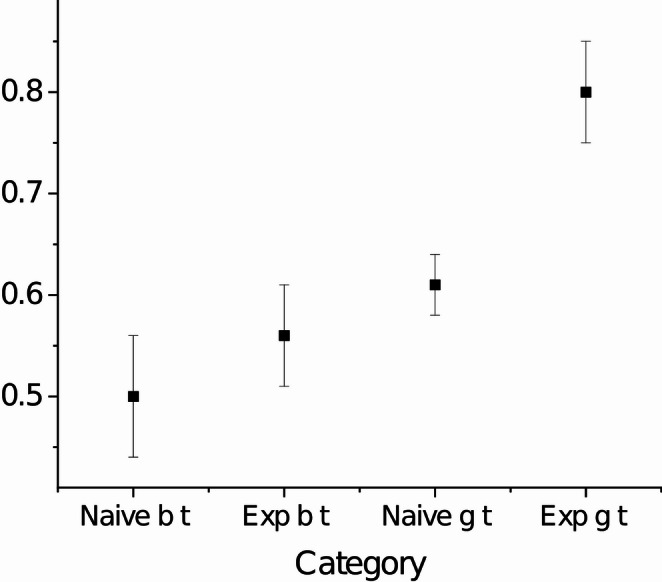
Proportion successful attempts out of the ten first in the transparent cylinder test for cylinder-naïve (*n* = 12) and cylinder-experienced (*n* = 11) blue tits (b t) and cylinder-naïve (*n* = 12) and cylinder experienced (*n* = 11) great tits (g t). The error bars are 95% confidence intervals. Data is from my own lab and have used in other figures in Isaksson et al. (2018 and Urhan et al. (2023)

To test if experience would affect the performance of the birds we positioned small transparent plastic tubes in some birds’ home cages and left them there for three days before testing started. This means that some birds had had prior access to transparent cylinder-like objects before being tested just like the ravens, while others were naïve in this respect. Interestingly, the great tits with cylinder experience increased their performance to 80 ± 0.05% while the experienced blue tits performed at a similar level as the naïve birds, 0.56 ± 0.05% with the dispersion measure being 96% CI (Fig. 2; Table 1). Hence, there was a learning effect in great tits but not in blue tits. This suggests that prior experience is an important factor in this type of testing which may make comparisons of cognitive ability between species and labs difficult. It also suggests that great tits are better observational learners than blue tits.

In most comparisons of spatial memory ability in hoarding and non-hoarding parids it has been shown that hoarding species perform better in spatial memory tests (Krebs et al. 1990; Healy et al. 1994). These tests, however, concerned caching like memory, for example observational memorization of food items positioned beforehand in caching holes covered by glass (Krebs et al. 1990). When it comes to memorization of more complex spatial memory tasks great tits may instead outperform hoarding parids. When caged great and marsh tits were allowed to observe other marsh tits storing in an aviary, great tits were much better than marsh tits at remembering caching locations afterwards (Brodin and Urhan 2014a, b; 2015; Urhan et al. 2017). This further supports the assumption that this species is an especially skilled observational learner.

**Table 1:** Two-sample t-tests for differences in the average proportional success between the bird categories in figure 2. The values have been arcsine square-root transformed before testing.

Categories compared	t	P	df
Naïve b t / Naïve g t	1.53	0.141	23
Exp b t / Exp g t	3.43	0.003	23
Naïve b t / Exp b t	0.78	0.443	23
Naïve g t / Exp g t	3.27	0.004	23

Len Howard was a British musician that formed a close relationship with her garden birds for many years. She lived in a small village in southernmost England where she kept her house, appropriately named bird cottage, open to small birds the whole year round. Even if she was not a scientist, few persons would know the individual garden birds as well as she did.

On cognitive ability in small birds she wrote “Watching birds closely and intimately, there are continual actions that cannot be accounted for by instinct and automatic reaction, but intelligence varies much with the individual as well as the species, and of the birds with which I am intimately acquainted, great tits reach the highest level of intelligence, consequently also of individuality within the species (Howard 1952). To me it seems as if great tits can specialize in almost anything that will produce food to their liking. Still, specific individuals or flocks will frequently differ in which types of specialized behaviours they use. There are only some birds that drum on bee hives, others that kill hibernating bats (Estók et al. 2009), yet others that kill smaller birds at bird feeders and as far as I know only one individual that have mastered to manufacture and use tools (Johnsson and Brodin 2019). I have spent many years working with hoarding parids in the forest and the foraging behaviour in these species seems more uniform with less variation between individuals (Brodin 1994a, b, 2005; Brodin et al. 1994; Brodin and Ekman 1994). As opposed to the innovative and curios nature of great tits and to some degree the blue tit the poecilid species may almost be considered as neophobic (Mönkkönen and Koivula 1993).

## Competitive release in North American chickadees?

The North American chickadees and the Eurasian willow and marsh tits are very closely related and belong to the same parid genus, *Poecile*. Also, they look very similar and have similar calls and behave very similarly (Brodin 2005). In small permanent flocks of two to six individuals they will constantly be on the move, frequently up in the trees, scouring their large year-round territory for food. When they encounter a food item they will either eat it directly or store it for future use.

When it comes to behaviour in anthropogenic habitats, however, the picture is very different. In Europe the great and blue tits are especially well adapted to these habitats whereas the poecilids appear not to be. In the reports that I have received as answers to my public calls for interesting foraging behaviours in parids 90% are on great tits and most of the remaining on blue tits (Johnsson and Brodin 2019; Brodin 2025). For poecilids I have received only one single report of “smart” foraging behaviour. A willow tit appeared to make false caches, i.e. behave like if it was caching without leaving the food item in the site if it was observed by potential cache thieves like great tits. This report was sent to me by an amateur ornithologist, and if it is correct this would be the first report of this deceptive behaviour in parids.

The opening of milk bottle caps that I discussed above may be acquired by at least two different cognitive abilities, first innovation by an individual that investigates a milk bottle and then subsequent observational learning by other individuals. According to Fisher and Hinde (1949) there were observations of 246 of blue tits doing this and 142 of great tits. For poecilids, they received only one report, of a marsh tit, showing this behaviour. Marsh tits are common in Britain, and they are frequently seen in gardens. The rareness of the behaviour in this species suggests that blue and great tits are much quicker at discovering and taking advantage of anthropogenic food sources than poecilids.

The poecilid species of which I have most personal experience of is the willow tit. Between 1989 and 1995 I studied hoarding behaviour in this species both in the field and in the lab. I still work on a population near Arvidsjaur in Swedish Lapland (65.5921 N, 19.1802 E). If I hang a bird feeder up in a new location in the forest it may take months before local willow tits start eating from it. This, however, only occurs if there are no great tits around. These will investigate a novel object like a feeder when they spot it. When they start taking sunflower seeds from it this will lure other tit-species, such as willow and Siberian tits to do so too. This has been observed not only to me, but also by Jan Ekman, my supervisor as a PhD student.

Judged from the literature and my experiences of black-capped chickadees (Brodin 2005) North American poecilds are much better adapted to anthropogenic habitats. In her book *The genius of birds* Ackerman (2016) describes the black-capped chickadee as “…acrobatic in its aptitudes, curious, intelligent and opportunistic, with a remarkable memory.” She continues “…they excel at discovering and exploiting new sources of food.” “…in winter they’ll eat bees, roosting bats, tree sap, and dead fish.” These behaviours are exactly what I would describe as exclusive for great tits in Europe. Never once have I experienced, heard or read about anything like this in willow or marsh tits. Not even the blue tit has ever been reported killing bees or roosting bats in the way great tits do.

Adaptation to anthropogenic habitats may occur also in other species of chickadees. Vladimir Pravosudov and his coworkers has worked on the mountain chickadee for many years. They describe birds from populations that live near human cities as “successful city slickers” (Kozlovsky et al. 2017). Compared to individuals from the natural montane forest habitat, birds near cities explored novel habitats faster, were better problem solvers and had better long-term memory retention.

What is then the difference between the North American and European poecilids that appear to be almost identical? I think it is the mainly the presence of great tit, but maybe also to some extent also the blue tit. The former species is almost twice the size (17–20 g) of poecilids (9–12 g) and it is not unusual that it uses its sharp beak to kill birds of poecilid size (Brodin 2025). The blue tit is of the same size as the poecilid species but aggressive and dominant over these (Gosler and Clement 2007). Among parids in Europe, the great tit and blue tits dominate gardens and city parks completely. The commonness of these two species will probably make anthropogenic habitats less favourable for poecilids. Both the marsh and willow tit will visit bird feeders in gardens near forests but only for occasional short bouts to take food.

As great and blue tits do not occur in North America this leaves the “anthropogenic parid niche” available for other species. As evident from the mountain chickadee example above there has been and may be ongoing selection for such adaptations. Without any aggressive great and blue tits gardens and parks may thus be a favourable habitat for North American poecilids.

In conclusion there are two distinctive wintering strategies in the parid family even though the species I discuss are closely enough related so that they until recently were considered to be members of the same genus, *Parus*. As small birds are very energetically stressed in winter, selection for optimal foraging strategies will be very strong in them. As the brain is an energetically expensive organ there is probably also a trade-off between cognitive specializations in different parts of it.

## Data Availability

No datasets were generated or analysed during the current study.

## Footnotes

1 Youtube https://www.youtube.com/watch?v=qFgoBVaj23I. The story about the deceptive alarm call starts after 2 minutes and 23 seconds.
